# Neurogenesis-dependent antidepressant-like activity of *Hericium erinaceus* in an animal model of depression

**DOI:** 10.1186/s13020-021-00546-8

**Published:** 2021-12-07

**Authors:** Pit Shan Chong, Chi Him Poon, Jaydeep Roy, Ka Chun Tsui, Sze Yuen Lew, Michael Weng Lok Phang, Rachael Julia Yuenyinn Tan, Poh Guat Cheng, Man-Lung Fung, Kah Hui Wong, Lee Wei Lim

**Affiliations:** 1 Neuromodulation Laboratory, School of Biomedical Sciences, Li Ka Shing Faculty of Medicine, The University of Hong Kong, 21 Sassoon Road, Hong Kong SAR, China; 2 Department of Anatomy, Faculty of Medicine, Universiti Malaya, 50603 Kuala Lumpur, Malaysia; 3 Institute of Biological Sciences, Faculty of Science, Universiti Malaya, 50603 Kuala Lumpur, Malaysia; 4 Ganofarm R&D SDN BHD, 01-01, SKYPOD SQUARE, Persiaran Puchong Jaya Selatan, Bandar Puchong Jaya, 47100 Puchong, Selangor Malaysia

**Keywords:** *Hericium erinaceus*, Depression, Neurogenesis, Anti-neuroinflammatory, Hippocampus

## Abstract

**Background:**

Depression is a severe neuropsychiatric disorder that affects more than 264 million people worldwide. The efficacy of conventional antidepressants are barely adequate and many have side effects. *Hericium erinaceus* (HE) is a medicinal mushroom that has been reported to have therapeutic potential for treating depression.

**Methods:**

Animals subjected to chronic restraint stress were given 4 weeks HE treatment. Animals were then screened for anxiety and depressive-like behaviours. Gene and protein assays, as well as histological analysis were performed to probe the role of neurogenesis in mediating the therapeutic effect of HE. Temozolomide was administered to validate the neurogenesis-dependent mechanism of HE.

**Results:**

The results showed that 4 weeks of HE treatment ameliorated depressive-like behaviours in mice subjected to 14 days of restraint stress. Further molecular assays demonstrated the 4-week HE treatment elevated the expression of several neurogenesis-related genes and proteins, including doublecortin, nestin, synaptophysin, brain-derived neurotrophic factor (BDNF), tropomyosin receptor kinase B (TrkB), phosphorylated extracellular signal-regulated kinase, and phosphorylated cAMP response element-binding protein (pCREB). Increased bromodeoxyuridine-positive cells were also observed in the dentate gyrus of the hippocampus, indicating enhanced neurogenesis. Neurogenesis blocker temozolomide completely abolished the antidepressant-like effects of HE, confirming a neurogenesis-dependent mechanism. Moreover, HE induced anti-neuroinflammatory effects through reducing astrocyte activation in the hippocampus, which was also abolished with temozolomide administration.

**Conclusion:**

HE exerts antidepressant effects by promoting neurogenesis and reducing neuroinflammation through enhancing the BDNF-TrkB-CREB signalling pathway.

**Supplementary Information:**

The online version contains supplementary material available at 10.1186/s13020-021-00546-8.

## Introduction

Major depressive disorder is a common mental illness that affects more than 264 million people of all ages worldwide [1]. The symptoms of major depressive disorder include appetite or sleep changes, fatigue, inhibited motivation, feelings of helplessness and hopelessness, loss of interest, self-loathing, and problems with concentrating and decision making [2–4]. Chronic symptoms of depression can affect psychosocial functioning and can lead to severe emotional, behavioural and physical health problems, and even suicide [2, 5]. Depressive disorder is one of the leading causes of the global burden of disease [1]. There are several proposed hypotheses for the pathophysiology of depression, including genetic factors, psychosocial stress, stress hormones, cytokines, monoamine deficiency (including serotonin, norepinephrine and dopamine), altered glutamatergic and GABAergic neurotransmission, and neurogenic and neurotrophic factors that affect brain volume [6]. Various classes of antidepressants have been developed to target different types of depression. Although many conventional antidepressants are available, up to 30% of patients with major depressive disorder fail to respond to their first prescribed antidepressant and need to be switched to another antidepressant [7]. The reasons for treatment failure often involve suboptimal treatment response or intolerable side effects, including weight gain or sexual dysfunction [8, 9]. Therefore, novel antidepressants are needed that have higher efficacy but less side effects.

Complementary and alternative medicines can be cost-effective treatments for depressive disorders and usually have less side effects [10]. *Hericium erinaceus* (HE) is a culinary and medicinal mushroom that has been reported to have significant beneficial health effects, including immunomodulatory [11], antitumour [12, 13], antidiabetic [14], antimicrobial [13], antioxidative [15], antihyperglycaemic [16], and hypolipidemic [17] activities. Furthermore, increasing evidence has shown that HE can promote positive nerve and brain health. HE has been shown to possess pharmacological activity that can improve neurological conditions, including cognitive impairment [18], Alzheimer’s disease [19], and Parkinson’s disease [20]. Recently, HE was shown to ameliorate depressive-like symptoms in both preclinical [21–23] and clinical studies [24–28]. Several pathways have been reported to be involved in the antidepressant effects of HE, including anti-inflammatory pathways [21], neurogenic and neurotrophic modulation [22, 23], and monoamine modulation [23]. The antidepressant effects of HE can be attributed to various bioactive compounds that can have diverse beneficial functions. Several bioactive compounds extracted from HE have been shown to stimulate the synthesis of nerve growth factor (NGF) and promote neurite outgrowth, which have a role in alleviating depressive-like symptoms [26, 29–35]. Furthermore, an in vivo study reported that HE enriched with erinacine A could modulate BDNF signalling, and enhance serotonin, dopamine, and noradrenaline expression levels [23]. These studies strongly suggest the antidepressant effects of HE are mediated through a neurogenesis-dependent mechanism*.*

Despite evidence showing that HE can potentially alleviate depressive-like symptoms [21, 28], the antidepressant-like effects of natural HE have yet to be examined in an animal model of depression. Therefore, this study aims to investigate the antidepressant-like effects of HE extracts in a chronic restraint stress (CRS) animal model of depression, and to elucidate the potential neurogenesis mechanisms. We hypothesised that HE would reduce depressive-like behaviours in the animal model of depression through a neurogenesis-dependent pathway.

## Experimental section

### Subjects

Male C57BL/6 mice (8–10 weeks, n = 76) were housed under controlled conditions (25–27°C temperature and 60–65% humidity) in a 12-h light/dark cycle. Food and water were available ad libitum. All animal procedures were approved by the Committee on the Use of Live Animals in Research (CULATR No. 4495-17), the University of Hong Kong. The animal model of depression was induced by CRS and naïve animals were used as the control.

### Extraction and nutritional composition of H. erinaceus

*Hericium erinaceus* standardised aqueous extract (NevGro®, Batch No. 7H2308X, Ganofarm R&D Sdn Bhd, Tanjung Sepat, Selangor, Malaysia) was used in this study. Briefly, the extract was prepared from fresh fruiting bodies of HE boiled in reverse osmosis water for 4 h, filtered, concentrated, and spray-dried. The aqueous extract consisted of 20.66% beta 1,3–1,6 glucan and 0.17% adenosine (Nova Laboratories Private Limited, Sepang, Selangor, Malaysia). Total glucan and α-glucan were quantified by a β-glucan assay kit (Megazyme International, Wicklow, Ireland). Adenosine content was analysed and measured by high-performance liquid chromatography (HPLC) using an in-house method (Nova Laboratories Private Limited, Sepang, Selangor, Malaysia) [36].

### Extraction, isolation, and identification of H. erinaceus compounds

*Hericium erinaceus* was macerated and extracted with 95% ethanol. The extract was concentrated *in vacuo* and subjected to isolation methods based on size exclusion and polarity chromatography. In the size exclusion-based method, the ethanol extract was fractionated using gel permeation chromatography (Sephadex LH-20, GE Healthcare, Uppsala, Sweden) with methanol (MeOH) into 10 fractions, from which compound **1** was derived from the 8th fraction. In the polarity-based method, the ethanol extract was subjected to liquid–liquid partitioning between n-butanol and water. The n-butanol fraction was fractionated using preparative radial chromatography with silica gel (Silica gel 60 PF_254_, Merck, Darmstadt, Germany). The solvent system used in preparative radial chromatography was chloroform (CHCl_3_) and 1–15% MeOH-CHCl_3_. The preparative radial chromatography procedure was repeated for three runs to give 10 to 13 fractions. The 6th fraction from the first run and the 5th fraction from the second and third runs were combined for further processing by preparative radial chromatography with silica gel and mobile phase of ether (Et_2_O) with an increasing MeOH gradient. Compound **2** was obtained from the 4th–6th fractions (8.6 mg) [37] and a medium polar compound **3** was obtained from the 2nd and 3rd fractions (3 mg). Additional file 1: Fig. S1B shows the steps involved in the isolation of the compounds. The compounds were analysed using spectroscopic methods: ^1^H and ^13^C nuclear magnetic resonance (NMR) spectra were recorded in MeOH-_d4_ on an FT-NMR Avance III 600 MHz (Bruker, Massachusetts, USA), high-resolution electrospray ionisation mass spectrometry (HRESIMS) data were obtained on an Agilent 6530 Q-TOF mass spectrometer (Agilent Technologies, California, USA), Ultraviolet (UV) spectra were obtained on a Shimadzu UV-2600 spectrophotometer (Shimadzu, Kyoto, Japan), and Infrared (IR) spectra were recorded on a Spectrum 400 FT-IR/FT-FIR spectrophotometer (PerkinElmer, Massachusetts, USA) [37, 38].

### Determination of total polyphenol content

HE was dissolved in 80% methanol (MeOH) at a ratio of 1:4 (w/v) with shaking for 30 min. The extract was centrifuged at 6500 rpm for 15 min at 4 °C in a refrigerated centrifuge (Sorvall ST 16R; Thermo Fisher Scientific, Waltham, Massachusetts, USA). The supernatant (10 µL) or gallic acid standard solution was mixed with 790 µL double-distilled water (ddH_2_O) and 5 µL Folin-Ciocalteu reagent. After 1 min, 150 µL sodium carbonate was added to the mixture and incubated for 2 h at room temperature in the dark. Absorbance was measured at 750 nm using a spectrophotometer (Eppendorf BioSpectrometer basic; Eppendorf, Hamburg, Germany) with gallic acid as the reference standard. Total polyphenol content was expressed as milligram gallic acid equivalent per gram of extract (mg GAE/g) [39, 40].

### Determination of total flavonoid content

HE was dissolved in 80% methanol (MeOH) at a ratio of 1:4 (w/v) with shaking for 30 min. The extract was centrifuged at 6500 rpm for 15 min at 4 °C in a refrigerated centrifuge (Sorvall ST 16R; Thermo Fisher Scientific, Waltham, Massachusetts, USA). The supernatant (250 µL) was mixed with 1250 µL ddH_2_O and 75 µL 5% sodium nitrite (NaNO_2_) at room temperature. After 5 min, 150 µL 10% aluminium chloride (AlCl_3_) was added into the mixture and incubated for 5 min at room temperature. After incubation, 500 µL 1 M sodium hydroxide (NaOH) and 275 µL ddH_2_O were added to the mixture. Absorbance was measured at 510 nm using a spectrophotometer (Eppendorf BioSpectrometer basic; Eppendorf, Hamburg, Germany) with catechin as the reference standard. Total flavonoid content was expressed as milligram catechin equivalent per gram of extract (mg CE/g) [41, 42].

### Determination of total antioxidant capacity

HE was dissolved in 80% methanol (MeOH) at a ratio of 1:4 (w/v) with shaking for 30 min. The extract was centrifuged at 6500 rpm for 15 min at 4 °C in a refrigerated centrifuge (Sorvall ST 16R; Thermo Fisher Scientific, Waltham, Massachusetts, USA). The supernatant (5 µL) was mixed with 95 µL 80% MeOH in 1000 µL reagent (0.6 M sulphuric acid, 28 mM sodium phosphate, and 4 mM ammonium molybdate) and incubated for 90 min at 95 °C in the dark. After incubation, the mixture was cooled to room temperature and absorbance was measured at 695 nm using a spectrophotometer (Eppendorf BioSpectrometer basic; Eppendorf, Hamburg, Germany) with ascorbic acid as the reference standard. Total antioxidant capacity was expressed as microgram ascorbic acid equivalent per gram of extract (mg AAE/g) [40, 43].

### DPPH free radical scavenging assay

A 20 µg/mL 2,2-diphenyl-1-picrylhydrazyl (DPPH) solution was first prepared in 99.8% ethanol (EtOH). Next, 2 mL DPPH solution was added to 1 mL HE dissolved in ddH_2_O and incubated for 20 min at room temperature in the dark. Absorbance was measured at 517 nm using a multimode plate reader (EnSpire™ 2300; PerkinElmer, Waltham, Massachusetts, USA). The scavenging activity was calculated using the following formula: % Activity = [1− (sample absorbance / blank absorbance)] × 100. The scavenging activity was expressed as EC_50_ (mg/mL), which is the effective concentration at which 50% of DPPH radicals are scavenged [44]. Ascorbic acid was used as the positive control.

### Ferric reducing antioxidant power (FRAP) assay

The FRAP reagent was prepared by mixing 300 mM acetate buffer, 20 mM ferric chloride hexahydrate (FeCl_3_.6H_2_O), and 10 mM tripyridyltriazine (TPTZ) in ddH_2_O. Next, 25 µL HE in ddH_2_O was mixed with 175 µL FRAP reagent and incubated for 4 min at room temperature. Absorbance was measured at 593 nm using a multimode plate reader (EnSpire™ 2300; PerkinElmer, Waltham, Massachusetts, USA) with ferrous sulphate (FeSO_4_.7H_2_O) as the reference standard. The change in absorbance was calculated by the following formula: Sample absorbance – (sample blank absorbance + reagent absorbance). The FRAP value was expressed as micromole ferrous sulphate equivalent per gram of extract (µmol FeSO_4_.7H_2_O equivalents/g) [45]. Ascorbic acid was used as the positive control.

### Screening for gene targets of HE, depression, and HE-depression overlap

HE contains Hericenone class compounds, Erinacine class compounds, ergosterol peroxide, and cerevisterol [28]. Additionally, our previous findings indicated that HE also contains adenosine and herierin III [37]. We used PubChem database (http://pubchem.ncbi.nlm.nih.gov) to search for corresponding gene targets of the compounds in HE. The gene ID and the gene name were retrieved by the subsequent procedures. For depression-related gene search, we performed the gene-target search using DisGeNET (http://www.disgenet.org), a database of gene-disease associations. We used the search term “Mental Depression” (UMLS CUI:C0011570) for the retrieval of gene targets. A total of 1478 of genes were initially returned, of which 261 genes remained after applying a filter of Score_gda_ $\ge$ 0.1 and EI_gda_ = 1. We then compared the gene lists from PubChem and DisGeNET to select replicated genes for the further downstream analyses.

### Network construction, gene ontology and KEGG pathway enrichment analysis

A compound-target network based on the PubChem results was constructed with Cytoscape V3.8.2, a software for network visualization. To examine the association between HE and depression at the genome level, gene ontology (GO) enrichment and Kyoto Encyclopedia of Genes and Genomes (KEGG) pathway analysis were performed in R (Version 2021.09.0) using the Bioconductor package (http://www.bioconductor.org/). All HE-depression overlapped genes were first converted to EntrezID. Genes were then annotated with ClusterProfiler and “Homo sapiens” genome library for the corresponding GO terms, and the results were visualized by ggplot2. The GO analysis consisted of three aspects: Biological Process (BP), Cellular Component (CC), and Molecular Function (MF). Only the top 10 results with p-value $\le$ 0.01 in each aspect were enriched and visualized. The HE-depression overlapped genes were also analysed by KEGG to examine the relationship of HE and depression in terms of the molecular pathways. Results from GO analysis and KEGG are presented in the dotplot and pathview diagrams.

### Experimental design and drug administration

The model of depression was generated by subjecting animals to CRS with immobilisation for 6 h per day, continuously for a total of 14 days. Animals received daily injection of HE at experimental doses of 10 and 25 mg/kg or 0.9% saline intraperitoneally for 4 weeks. The control non-CRS animals were injected with 0.9% saline. On days 8, 10, and 12, Bromodeoxyuridine (BrdU; 150 mg/kg; Sigma-Aldrich, Missouri, USA) was intraperitoneally injected to monitor cell proliferation and neurogenesis in the animal brain. Temozolomide (TMZ) was used to block hippocampal neurogenesis and was injected in the first 3 weeks (days 1, 3, 5, 8, 10, 12, 15, 17, 19, 22, 24, and 26). A battery of behavioural tests was applied: cage emergence test and novelty-suppressed feeding test to assess anxiety, and sucrose preference test and tail suspension test to evaluate depressive-like behaviours. For details of the experimental design, see Fig. 3A.

### Behavioural testing

All behavioural tests were performed as previously described with minor modifications [46]. For the cage emergence test, animals were individually placed in an aversive cage with light (450 Lux) shining on the cage. A grid was placed in the open cage to allow the animals to escape the cage. Their movement was recorded by video to determine the latency to escape from the cage. For the novelty-suppressed feeding test, an open field (40 × 40 × 40 cm) was used to evaluate the animal’s aversion to eating in a novel environment. The animal was deprived of food but allowed access to water for 24 h prior to testing. The animal was placed on the edge of a brightly lit open field with a petri dish of rodent chow located in the centre of the field. Latency to feed was recorded within 10 min to assess the level of anxiety. For the sucrose preference test, animals were housed individually and pre-exposed to 1% sucrose solution without water for 1 h. Prior to testing, animals were restricted to food and water for 14 h. A bottle of pre-weighed 1% sucrose solution and a bottle of pre-weighed water were provided to the animal for 2 h. Sucrose intake and water intake were recorded by weight. The sucrose preference index as a measure of stress-induced anhedonia was calculated using the following formula: Sucrose preference (%) = (sucrose intake) / (water intake + sucrose intake) × 100%. For the tail suspension test, the animal was suspended in a suspension box (40 cm high) by taping the tail to the top of the box using adhesive tape. Their movement was video recorded to measure the immobility time during the 5-min test.

### Tissue processing

The animals were euthanised with sodium pentobarbital (Dorminal, Alfasan International BV, Woerden, Holland). Animals were decapitated and their extracted brains were immediately frozen in liquid nitrogen and stored at − 80 °C for the gene and protein expression studies.

### Real-time PCR

The dorsal hippocampus was dissected to examine the expression levels of neurogenesis-related genes. Real-time PCR was performed according to previously published methodology [47, 48]. Total RNA was isolated from the hippocampus using TRIZOL (Life Technologies, Carlsbad, USA) and converted into cDNA using a cDNA synthesis kit (Takara Bio Inc., Shiga, Japan). Quantitative real-time PCR (qPCR) of neurogenesis- and neuroplasticity-related genes including *brain-derived neurotrophic factor* (*Bdnf*), *tropomyosin receptor kinase B (Trkb), neuronal nuclei (Neun), doublecortin* (*Dcx*), *synapsin* (*Syn*), *nestin* (*Nes*), *cAMP response element-binding protein (Creb),* and *postsynaptic density-95 (Psd-95)* were performed using the StepOnePlus Real-Time PCR system (Thermo Fisher Scientific, Massachusetts, USA) with SYBR Green quantitative PCR mix (Applied Biosystems, Warrington, UK). The primer sequences used in this study can be found in Table 1. The relative expression was calculated as the relative quantification normalised to the reference *glyceraldehyde 3-phosphate dehydrogenase* (*Gapdh)* gene using the ratio 2^−ΔΔC^_T_ method [47, 49].

**Table 1:** The primer sequences used in the real-time quantitative PCR

Gene	5′–3′ primer sequence
*Bdnf *[104]	Forward: TGGCTGACACTTTTGAGCAC Reverse: AAGTGTACAAGTCCGCGTCC
*TrkB *[105]	Forward: CCTCCACGGATGTTGCTGAC Reverse: GCAACATCACCAGCAGGCA
*NeuN *[106]	Forward: GAGGAGTGGCCCGTTCTG Reverse: AGGCGGAGGAGGGTACTG
*Dcx *[107]	Forward: ACACCCTTGATGGAAAGCAG Reverse: AGGACCACAAGCAATGAACA
*SYP *[108]	Forward: TGTGTTTGCCTTCCTCTACTC Reverse: TCAGTGGCCATCTTCACATC
*Nes *[109]	Forward: AGGCTGAGAACTCTCGCTTGC Reverse: GGTGCTGGTCCTCTGGTATCC
*Creb *[110]	Forward: CAGGGGTCGCAAGGATTGAAG Reverse: ATCGCCTGAGGCAGTGTACT
*Psd-95 *[111]	Forward: GACGCCAGCGACGAAGAG Reverse: CTCGACCCGCCGTTTG
*Gapdh *(47)	Forward: GTCGGTGTGAACGGATTTG Reverse: AATTTGCCGTGAGTGGAGTC

### Protein preparation and Western blot analysis

The dorsal hippocampus was homogenised with RIPA buffer containing protease and phosphatase inhibitors (Thermo Scientific, Rockford, Illinois, USA). The protein concentration was measured by Bio-Rad DC Protein Assay Kit (Bio-Rad, Hercules, California, USA). The samples were separated by 8–12% SDS-PAGE and transferred to PVDF membranes (Bio-Rad Laboratories, Hercules, California, USA) using a semi-dry electroblotting system. The membranes were blocked with 5% BSA in TBS-T (20 mM Tris–HCl, 150 mM NaCl, 0.1% Tween 20) for 1 h at room temperature. Blots were incubated at 4 °C overnight with respective primary antibodies, including TrkB, pTrkB (1:1000; Millipore, Massachusetts, USA), pCREB (1:500; Cell Signaling Technology, Inc., Beverly, Massachusetts, USA), CREB, ERK1/2, pERK1/2, GAPDH (1:1000; Cell Signaling Technology, Inc., Massachusetts, USA), and BDNF (1:1000; Abcam, Cambridge, Massachusetts, USA). Horseradish peroxidase-conjugated anti-rabbit immunoglobulin G antibody (Invitrogen, Thermo Fisher Scientific, Massachusetts, USA) was added for 1 h at room temperature. Bound proteins were visualised by chemiluminescence kit (Bio-Rad Laboratories, Inc., Hercules, California, USA) and the relative protein expression level was normalised against GAPDH.

### Immunofluorescence staining

Animals were sacrificed and transcardially perfused with 4% paraformaldehyde. Brain tissue was extracted and post-fixed in 4% paraformaldehyde with cryoprotection in 15% and 30% sucrose solution until the tissues sank to the bottom. Brain tissues were snap-frozen in liquid nitrogen before storing in a -80 °C freezer. Coronal sections were obtained in a CryoStar NX50 Cryostat (Thermo Fisher Scientific, Massachusetts, USA). Brain sections were blocked with 3% H_2_O_2_ solution before pre-treating with 2 N HCl for 30 min at 37 °C. After blocking with 5% BSA for 30 min, sections were incubated with anti-BrdU (1:500; ab6326, Abcam, Cambridge, Massachusetts, USA) and anti-NeuN (1:1000; MAB377, Millipore, Massachusetts, USA) at 4 °C overnight. Sections were incubated with goat anti-rat IgG Dylight 488 (1:1000; ab150157, Abcam, Cambridge, Massachusetts, USA) and goat anti-mouse IgG Alexa Fluor 546 (1:1000; A-11003, Invitrogen, Thermo Fisher Scientific, Massachusetts, USA). Sections were counterstained with DAPI, and images were acquired using an Olympus BX53 Fluorescence microscope.

### Immunohistochemistry

Brain sections were obtained as described above. Brain sections were blocked with 0.5% H_2_O_2_ solution and 1% BSA and then incubated with anti-GFAP (1:1000; 561483, BD Biosciences, San Diego, California, USA) at 4 °C. Sections were incubated with biotinylated horse anti-goat IgG (1:1000; BA9500, Vector Laboratories, California, USA) and horse anti-mouse IgG (1:1000; BA-2000, Vector Laboratories). Sections were counterstained with haematoxylin and images were acquired using a bright-field microscope with cellSens imaging software (Olympus, Japan).

### Statistical analysis

Statistical analyses were performed using IBM SPSS Statistics 25. All data were screened for normal distribution. Results for antioxidant capacity, scavenging assay, and FRAP assay were analysed by independent sample t-test with *p* < 0.001 considered statistically significant. Data from behavioural tests, gene assays, Western blot analysis, and immunohistochemistry and immunofluorescence staining were analysed by one-way ANOVA with LSD post-hoc test for multiple detailed comparisons among CRS groups. Student’s t-test was used for the comparison between non-CRS control group and CRS + saline group to verify the effective induction of depressive behaviour. Dunnett’s multiple comparison was used for comparisons with the non-CRS control group. All data was presented as mean ± S.E.M. and *p* ≤ 0.05 was considered statistically significant.

## Results

### Phytochemical content and in vitro antioxidant activities of HE

Table 2 shows the phytochemical constituents and in vitro antioxidant activities of HE. The total polyphenol and flavonoid constituents were 2.26 ± 0.20 mg GAE/g and 0.73 ± 0.03 mg CE/g, respectively. The antioxidant activities as measured by total antioxidant capacity was 7.79 ± 0.43 mg AAE/g. The DPPH free radical scavenging activity (EC_50_) was 1.44 ± 0.05 mg/mL. The ferric reducing antioxidant power (FRAP) was 52.34 ± 4.82 µmol FeSO_4_.7H_2_O equivalents/g.

**Table 2:** Phytochemical content and in vitro antioxidant activity of HE

*H. erinaceus* / Ascorbic acid	Total polyphenol content (mg GAE/g)	Total flavonoid content (mg CE/g)	Total antioxidant capacity (mg AAE/g)	DPPH (EC_50_; mg/mL)	FRAP (µmol FeSO_4_.7H_2_O equivalents/g)
*H. erinaceus*	2.26 ± 0.20	0.73 ± 0.03	7.79 ± 0.43	1.44 ± 0.05^a^	52.34 ± 4.82^a^
Ascorbic acid	–	–	–	0.01 ± 0.00^b^	2648 ± 50.29^b^

Values are presented as mean ± SD. Means with different letter superscripts in the same assay are significantly different (*p* < 0.001)

*AAE* ascorbic acid equivalent, *CE* catechin equivalent, *DPPH* 2,2-diphenyl-1-picrylhydrazyl, *EC*_*50*_ half-maximal effective concentration, *FeSO*_*4*_*.7H*_*2*_*O* ferrous sulphate, *FRAP* ferric reducing antioxidant power, *GAE* gallic acid equivalent, *HE* *H. erinaceus* standardised aqueous extract

### Isolation and structural elucidation of HE compounds

**Fig. 1:**
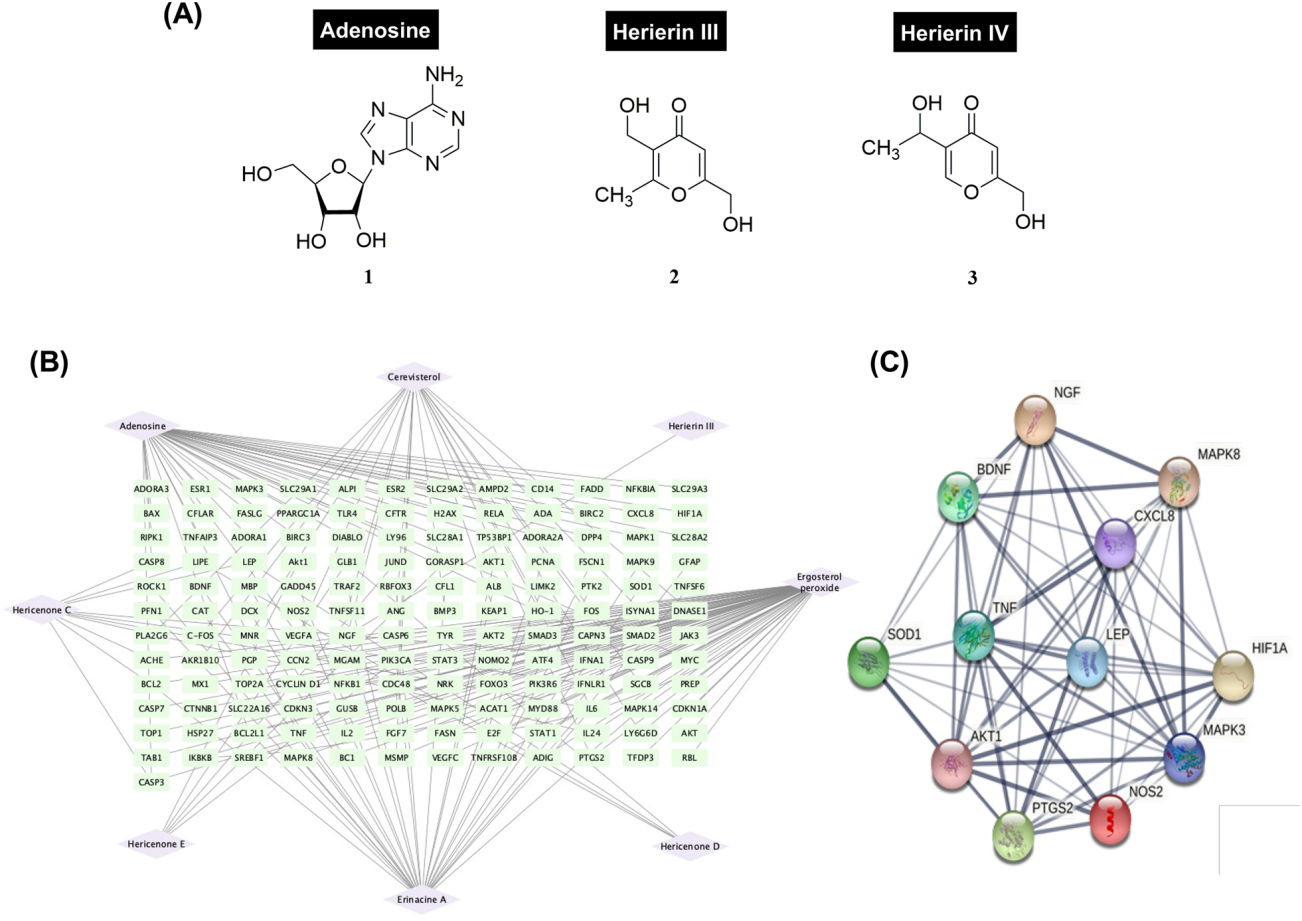
Structure of adenosine (**1**), herierin III (**2**), and herierin IV (**3**) (**A**). The compound-target network. The purple diamonds represent the 8 previously reported active compounds in HE. The green rectangles represent gene targets retrieved from PubChem database (**B**). Genes related to depression were retrieved from DisGeNET and compared with HE gene targets. Overlapped gene targets are shown (**C**)

Figure 1 shows the chemical structures of compounds isolated from HE, namely adenosine (**1**), herierin III (**2**) and herierin IV (**3**). Adenosine is a white powder and herierin III is a colourless oil [37]. Herierin IV (3 mg, 0.005%) was isolated as a colourless oil. The UV spectrum showed absorption peaks (${\lambda }_{\mathrm{max}}$) at 224.50 and 250.50 nm, suggesting a pyrone chromophore. The IR spectrum revealed peaks at 3348.05 and 1659.81 cm^−1^ due to the presence of OH group and unsaturated ketone functionalities, respectively. The HRESIMS showed an [M + H]^+^ peak at *m/z* 171.0658, revealing the molecular formula of herierin IV as C_8_H_10_O_4_. The complete NMR data assignments of herierin IV is summarised in Additional file 1: Fig. S1 and Additional file 2: Table S1.

### Prediction analysis of the pharmacological mechanism of HE based on network pharmacology

To probe the potential molecular mechanism of HE, we constructed a compound-target network based on previous findings that HE contains Hericenone class compounds, Erinacine class compounds, ergosterol peroxide, cerevisterol, adenosine, and herierin III [28, 37]. We obtained a total of 182 genes from 7 gene target lists. The resulting network had 154 nodes and 190 edges (Fig. 1B). Among the genes within the network, IL (degrees of freedom = 4), BCL2L1 (degrees of freedom = 3), BCL2 (degrees of freedom = 3), TNF (degrees of freedom = 3), CASP9 (degrees of freedom = 3), PIK3CA (degrees of freedom = 3), CASP3 (degrees of freedom = 3), and NGF (degrees of freedom = 3) were found to have the most connections with various compounds in HE, suggesting they are potential targets of HE. To establish an association between HE potential gene targets and depression, genes were retrieved from DisGeNET using the search term “Mental Depression”. The results were cross-referenced with the HE gene target list to produce a protein–protein interaction network of overlapped genes (Fig. 1C), which was used in the GO and KEGG analyses.

### GO and KEGG analyses predict the involvement of MAPK, neuroinflammation, and neurotrophin pathways in HE-depression gene targets

**Fig. 2:**
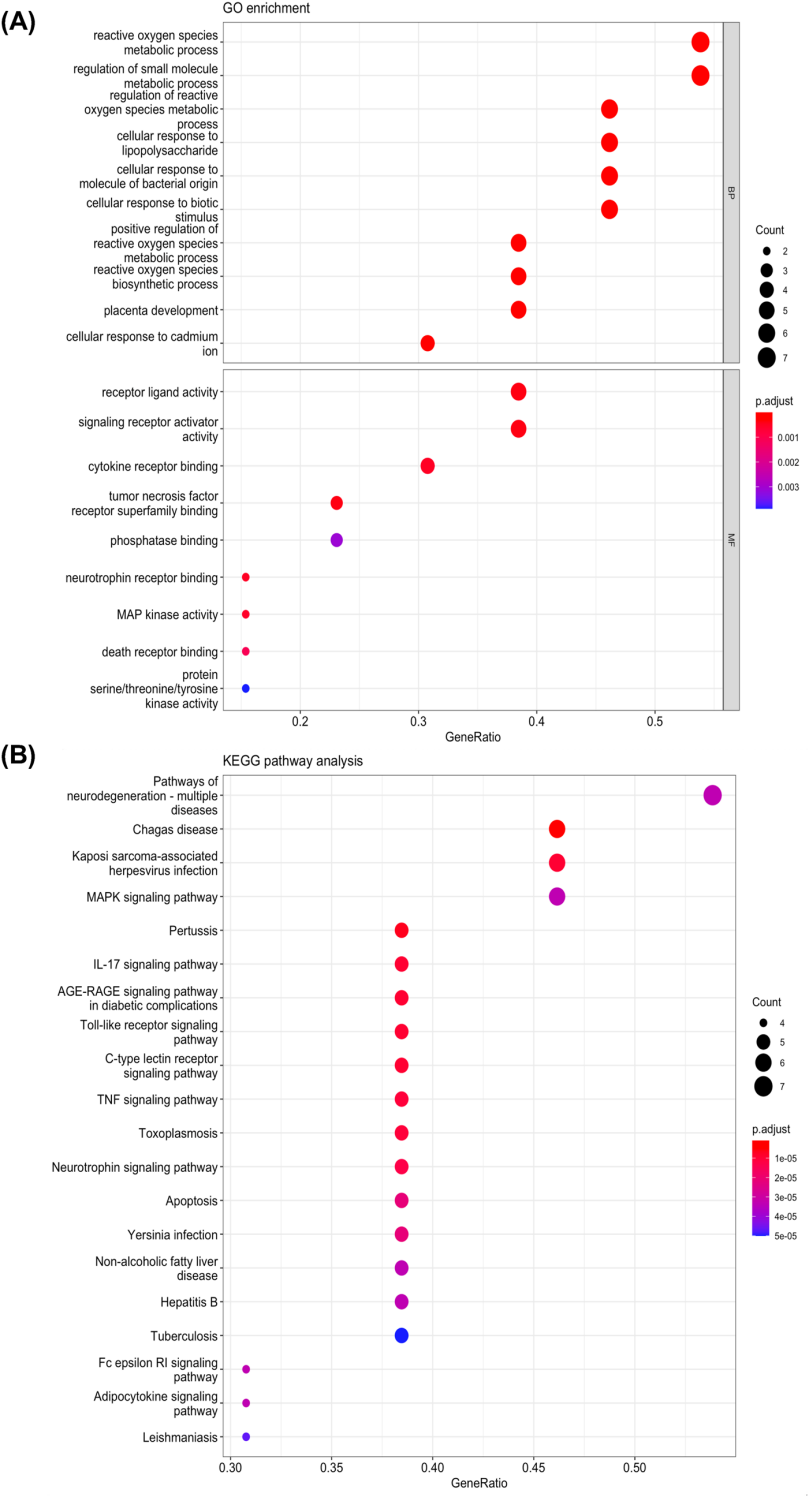
Bubble maps of GO annotation analysis (**A**) and KEGG pathway analysis (**B**). The Y axis shows the GO terms and the names of the enriched pathways. The area of the bubble represents the number of enriched genes. Adjusted p-value represented by a colour scale, with the statistical significance increasing from blue to red (blue represents low significance and red represents high significance)

We next performed GO annotation, enrichment, and KEGG pathway analyses of HE-depression overlapped genes. The STRING database was employed to assign genes under GO terms in three categories: biological process (BP), cellular component (CC), and molecular function (MF). The results of the GO analysis returned 428 entries related to BP, 0 entries related to CC, and 9 entries related to MF. The top 10 results with p-value $\le$ 0.01 from each category were selectively visualized, which showed pathways under BP consisted of metabolic processes and the regulation of reactive oxygen species, whereas pathways under MF consisted of receptor-ligand activity, signalling receptor activity, and cytokine receptor binding (Fig. 2A). These results demonstrate that HE may exert its effects through the modulation of oxidative stress and molecular components involved in neuroinflammation. We then performed pathway enrichment analysis and revealed that the most significantly enriched pathways included Chagas disease, Kaposi Sarcoma-associated herpesvirus infection, IL-17 signalling pathway, AGE-RAGE signalling pathway, TNF signalling pathway, neurotrophin signalling pathway, apoptosis, and MAPK signalling pathway (Fig. 2B). Given the previously reported associations of HE with MAPK signalling [50], neuroinflammation [51], and neurotrophins [52], we selected MAPK, IL-17, TNF and neurotrophin pathways for further visualization (Additional file 1: Figs. S2, S3).

### HE induces robust anxiolytic and antidepressant-like effects in the animal model of depression

**Fig. 3:**
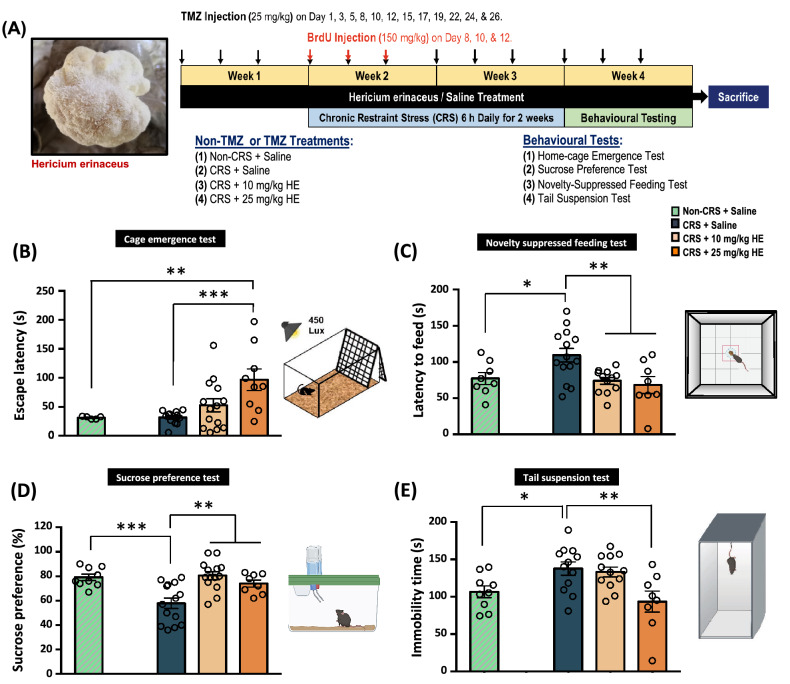
Schematic representation of the study of the effects of HE in the CRS model of depression (**A**). The assessment of anxiety-like behaviour by escape latency in the cage emergence test (**B**) and by latency to feed in the novelty suppressed feeding test. **C** The evaluation of depressive-like behaviour by percentage sucrose preference (**D**) and by immobility time in the tail suspension test (**E**). The results are presented as mean ± S.E.M. Indicators: *Significant difference between the CRS groups. *p ≤ 0.05. **p ≤ 0.01; ***p ≤ 0.001

To demonstrate the antidepressant effect of HE in the animal model of depression, a series of behavioural tests were performed to test the animals’ anxiety level and depressive-like behaviours (Fig. 3A). We first analysed the latency to emerge in the novel cage emergence test as a parameter of anxiety. One-way ANOVA revealed significant main effects on the novel cage emergence test (F _(3, 36)_ = 5.918, *p* = 0.002) (Fig. 3B). The analysis showed no significant differences between the CRS and non-CRS control group (t_(15)_ = 0.077, *p* = n.s.). Interestingly, the CRS group treated with 25 mg/kg HE exhibited significantly longer escape latency compared to the CRS + saline group (*p* < 0.001), indicating 25 mg/kg HE had an anxiolytic effect. To confirm the anxiolytic property of HE, we performed the novelty suppressed feeding test. One-way ANOVA showed significant main effects on the latency to feed among groups (F _(3, 38)_ = 5.279, *p* = 0.004) (Fig. 3C). Validating the effect of CRS on anxiety, the Student’s t-test demonstrated significant differences between the CRS + saline and non-CRS control groups (t_(20)_ = 2.316, *p* = 0.031). Further analysis confirmed that HE had an anxiolytic effect, as indicated by a significantly shorter latency to feed in both 10 mg/kg HE (*p* = 0.004) and 25 mg/kg HE (*p* = 0.003) groups compared to the CRS + saline group. The one-way ANOVA showed similar significant group effects in the sucrose preference test (F_(3, 41)_ = 9.537, *p* < 0.001) (Fig. 3D). The CRS + saline group exhibited lower sucrose preference as indicated by multiple comparisons compared with the non-CRS control group (t_(21)_ = -3.686, *p* = 0.001). Further analysis found both 10 mg/kg HE (*p* < 0.001) and 25 mg/kg HE (*p* = 0.009) groups had a significantly higher sucrose preference level compared with the CRS + saline group. The tail suspension test was then used to assess the animals’ depressive behaviour. One-way ANOVA showed significant main effects among the groups (F_(3, 37)_ = 5.023, *p* = 0.005) (Fig. 3E). The CRS + saline group exhibited a higher degree of depressive behaviour, as indicated by a significant increase in the immobility time (t_(19)_ = 2.515, *p* = 0.021) compared with the non-CRS control group. Specifically, multiple comparisons showed significantly shorter immobility time in the 25 mg/kg HE group (*p* = 0.004) compared with the CRS + saline group. These results showed that HE possesses anxiolytic and antidepressant effects, which validated the use of CRS as a viable protocol for inducing anxiety and depressive behaviours.

### HE administration enhances the expression of neuroplasticity-related genes

**Fig. 4:**
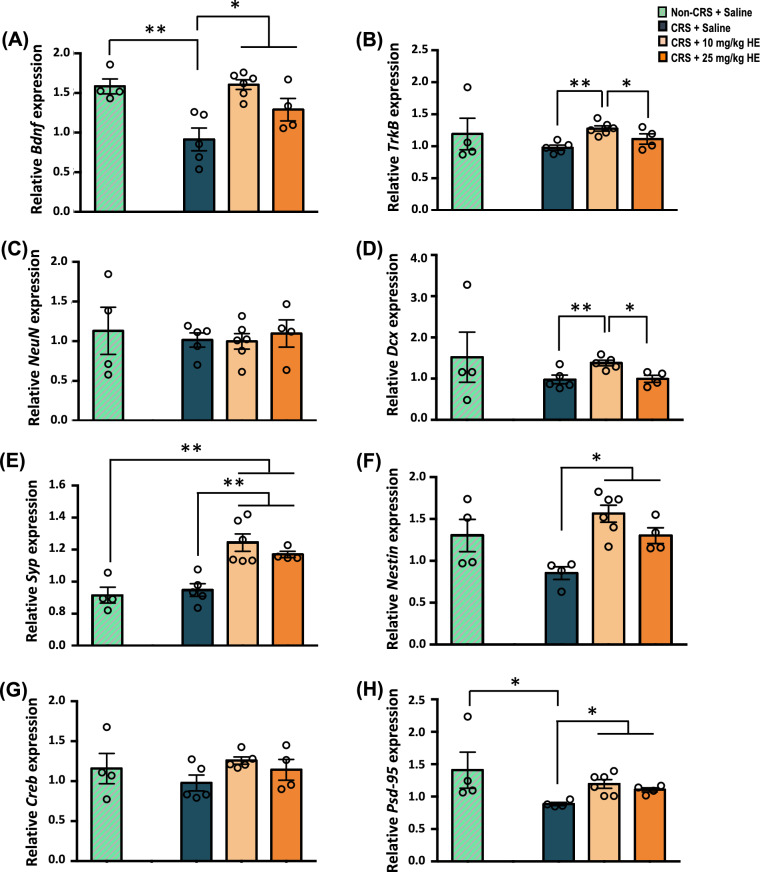
Effects of HE on the relative expression of neuroplasticity- and neurogenesis-related genes including *Bdnf* (**A**), *Trkb* (**B**), *NeuN* (**C**), *Dcx* (**D**), *Syp* (**E**), *Nes* (**F**), *Creb* (**G**), and *Psd-95* (**H**) in the dorsal hippocampus of non-CRS + saline, CRS + saline, CRS + 10 mg/kg HE and CRS + 25 mg/kg HE groups. BDNF was significantly downregulated in the CRS + saline group. Notably, 10 and 25 mg/kg HE increased the expressions of *Bdnf, Syp, Nes,* and *Psd-95*. Relative expression was calculated by normalising the relative quantifications to the reference gene *GAPDH* as a ratio of the 2^CT(reference) and 2^CT(test). *p ≤ 0.05. **p ≤ 0.01

Given the previously reported involvement of the hippocampus in the pathophysiology of depression [53], we selected the dorsal hippocampus to evaluate the expression of plasticity-related genes to identify the molecular effects of HE on depression. We found significant group effects on the gene expression of *Bdnf* (F_(2,12)_ = 10.213, *p* = 0.003), *Trkb* (F_(2,12)_ = 9.143, *p* = 0.004), *Dcx* (F_(2,11)_ = 6.810, *p* = 0.012), *Syp* (F_(2,12)_ = 12.227, *p* = 0.001), *Nes* (F_(2,11)_ = 13.728, *p* = 0.001), and *Psd-95* (F_(2,12)_ = 8.236, *p* = 0.007) (Fig. 4A, B, D, E, F, H). Further analysis revealed reduced *Bdnf* (*p* = 0.002) and *Psd-95* (*p* = 0.047) transcript expressions in the CRS + saline group compared with the non-CRS control group, and higher gene expressions of *Bdnf*, *Syp*, *Nes,* and *Psd-95* in both the 10 mg/kg HE group (*p* = 0.001; *p* < 0.001; *p* < 0.001; *p* = 0.002, respectively) and 25 mg/kg HE group (*p* = 0.047; *p* = 0.007; *p* = 0.012; *p* = 0.024, respectively) compared with the CRS + saline group. Interestingly, the 10 mg/kg HE group showed significantly higher expressions of *Dcx* and *Trkb* compared with the 25 mg/kg HE (*p* = 0.012; *p* = 0.048) and CRS + saline (*p* = 0.007; *p* = 0.001) groups. No significant differences were observed in transcript levels of *Neun* and *Creb* (Fig. 4C, G). These results suggest that HE possibly mediates its antidepressant effects by enhancing the expression of neuroplasticity-related genes, including restoring the *Bdnf* signalling impaired by CRS.

### HE increases the expression of neuroplasticity-related proteins

**Fig. 5:**
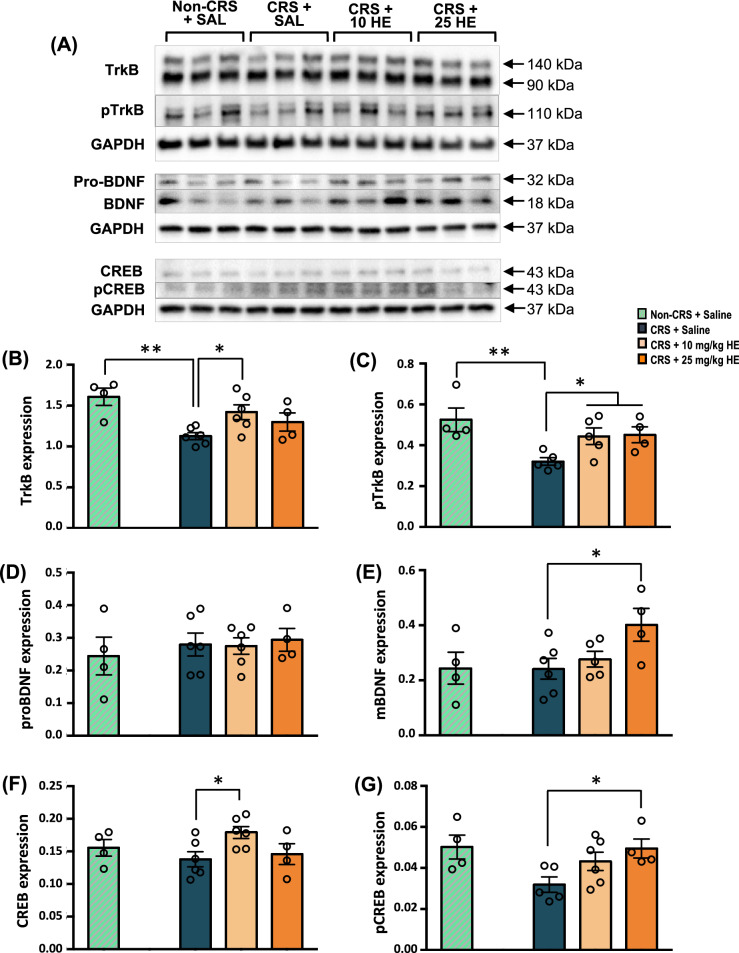
Western blot analysis of proteins associated with BDNF/TrkB signalling in dorsal hippocampal tissue from non-CRS + saline, CRS + saline, CRS + 10 mg/kg HE, and CRS + 25 mg/kg HE groups. Note, dividing lines represent cropped blots and white spaces represent cropped bands. Graphical representation of the effects of HE on the expression of neuroplasticity-related proteins (**A**) including TrkB (**B**), pTrkB (**C**), pro-BDNF (**D**), mature BDNF (**E**), CREB (**F**), and pCREB (**G**). The expression of target proteins was normalised to the expression of GAPDH. Note, there were significant increases in the expression of pTrkB, mBDNF, and pCREB proteins in the CRS + 25 mg/kg HE group compared with the CRS + saline group. *p ≤ 0.05. **p ≤ 0.01

To determine the molecular pathways that mediate the therapeutic effects of HE on depression, we studied changes in the activity of proteins contributing to synaptic plasticity in the dorsal hippocampus of HE-treated animals (Fig. 5A). To elucidate the molecular pathways in the neuroplasticity-related mechanisms, we first assessed the components of BDNF signalling and their involvement in the pathophysiology of depression [53]. One-way ANOVA revealed a marginally significant difference in the protein expression of TrkB (F_(2, 13)_ = 3.734, *p* = 0.052) and significant difference in pTrkB level (F_(2, 11)_ = 4.811, *p* = 0.032). Further analysis showed reductions in both TrkB (*p* = 0.003) and pTrkB (*p* = 0.007) protein levels in CRS + saline animals compared with non-CRS control animals (Fig. 5B, C). Compared with CRS + saline group, TrkB levels were significantly increased in the 10 mg/kg HE group, but not in 25 mg/kg HE group, although both 10 and 25 mg/kg HE enhanced pTrkB levels, indicating increased activation of the TrkB receptor. Next, we examined changes in the expression of pro-BDNF and its cleavage product mature BDNF (mBDNF), which interacts with TrkB and contributes to neuronal survival and plasticity [54]. Although no significant difference was found in the expression of pro-BDNF, we observed a marginally significant group effect on mBDNF levels (F_(2, 12)_ = 3.807, *p* = 0.052) (Fig. 5D, E). Consistently, we observed increased pTrkB level with higher mBDNF level in the CRS + 25 mg/kg HE group compared with the CRS + saline group. We also assessed the level of CREB and its phosphorylated form pCREB, a downstream molecule in BDNF-TrkB signalling [53]. We observed marginally significant differences in CREB (F_(2, 13)_ = 3.746, *p* = 0.052) and pCREB (F_(2, 12)_ = 3.854, *p* = 0.051) protein expression levels (Fig. 5F, G). The 10 mg/kg HE group, but not 25 mg/kg HE group, showed increased CREB protein levels in the dorsal hippocampus (*p* = 0.021). Compared with non-CRS control animals, CRS + saline animals showed reduced pCREB levels (*p* = 0.042), which were effectively restored by the administration of 25 mg/kg HE (*p* = 0.020)*.* These findings suggest an impaired BDNF-TrkB-CREB signalling pathway in animals receiving CRS, which could be effectively rescued by HE.

**Fig. 6:**
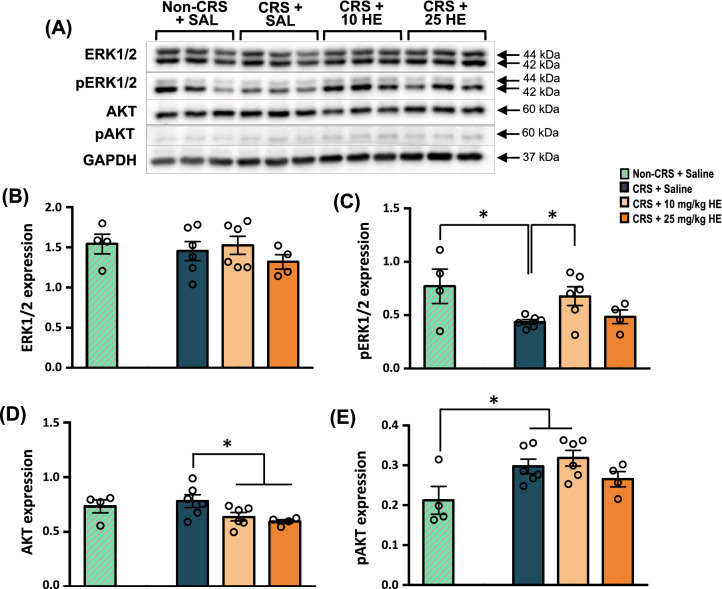
Western blot analysis of proteins associated with CREB phosphorylation in dorsal hippocampal tissue from non-CRS + saline, CRS + saline, CRS + 10 mg/kg HE, and CRS + 25 mg/kg HE groups. Note, dividing lines represent cropped blots and white spaces represent cropped bands. Blot images (**A**) and graphical representation of the effects of HE on the expression of neuroplasticity-related proteins including ERK1/2 (**B**), pERK1/2 (**C**), AKT (**D**), and pAKT (**E**). The expression of target proteins was normalised to the expression of GAPDH. Note, in CRS + 10 mg/kg HE group, there was a significant increase in pERK1/2 protein expression compared with the CRS + saline group and increase in pAKT compared to the non-CRS control group. *p ≤ 0.05

Given that 25 mg/kg HE was able to restore the reduced CREB phosphorylation, we examined the expression of proteins and the activation of protein kinases, including ERK1/2 [55] and AKT [56], which were previously reported to phosphorylate CREB (Fig. 6A). One-way ANOVA showed no significant difference in ERK1/2 protein level among groups (F_(2, 13)_ = 0.741, *p* = n.s.), indicating that CRS and HE did not affect the basal expression level of ERK1/2 (Fig. 6B). Interestingly, a significant group effect was observed on the phosphorylation of ERK1/2 (F_(2, 13)_ = 4.173, *p* = 0.040), in which a reduced pERK1/2 level was observed in CRS + saline animals compared with non-CRS control animals (*p* = 0.043), which was effectively rescued by 10 mg/kg HE (*p* = 0.016). However, further improvement was not observed with 25 mg/kg HE (Fig. 6C). On the other hand, a significant group effect was observed in the expression of AKT (F_(2, 13)_ = 4.43, *p* = 0.034), in which both 10 mg/kg (*p* = 0.037) and 25 mg/kg HE (*p* = 0.018) groups had reduced AKT protein levels compared with the CRS + saline group (Fig. 6D), whereas CRS increased AKT phosphorylation (p = 0.047). However, HE did not affect AKT phosphorylation (F_(2, 13)_ = 1.65, *p* = n.s.) (Fig. 6E).

### HE promotes hippocampal neurogenesis in an animal model of depression

**Fig. 7:**
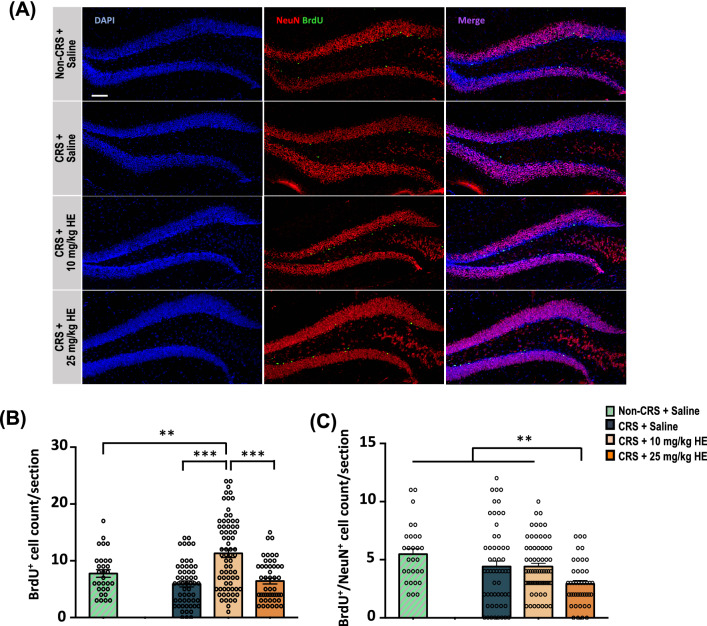
Immunofluorescence staining of BrdU and NeuN in the DG reveals a dosage-dependent effect of HE treatment on neurogenesis. Scale bar: 100 µm (**A**). Note, there was a significant increase in BrdU^+^ cells in the DG of CRS animals receiving 10 mg/kg HE, but not 25 mg/kg HE (**B**). Double labelling of BrdU and NeuN showed deceased neuronal maturation in the CRS + 25 mg/kg HE group (**C**). **p ≤ 0.01; ***p ≤ 0.001

Based on the results from the gene and protein assays that demonstrated enhancement in neurogenesis-related molecular pathways and the results from the behavioural screening that supported a neurogenesis-dependent mechanism of the antidepressant-like effects of HE, we performed further morphological studies on the dentate gyrus (DG) of the hippocampus, a region where adult neurogenesis mainly occurs [57] (Fig. 7A). Quantification of neurogenesis marker BrdU^+^ cells in the DG revealed a significant group effect (F_(2,170)_ = 23.11, *p* < 0.001). Further analysis showed 10 mg/kg HE, but not 25 mg/kg HE, had a remarkable neurogenic effect (Fig. 7B). To determine the effect of HE treatment on neural differentiation, we examined the co-localisation of BrdU with mature neuronal marker NeuN in the DG. One-way ANOVA on CRS animals showed a significant group effect in the DG (F_(2,160)_ = 4.95, *p* = 0.008). Interestingly, animals receiving 25 mg/kg HE had significantly lower BrdU^+^/NeuN^+^ cell count in the DG (Fig. 7C). Taken together, these results provide morphological evidence supporting that 10 mg/kg HE has higher efficacy in promoting hippocampal neurogenesis.

### Blockade of neurogenesis abolishes the anxiolytic and antidepressant-like effects of HE in an animal model of depression

**Fig. 8:**
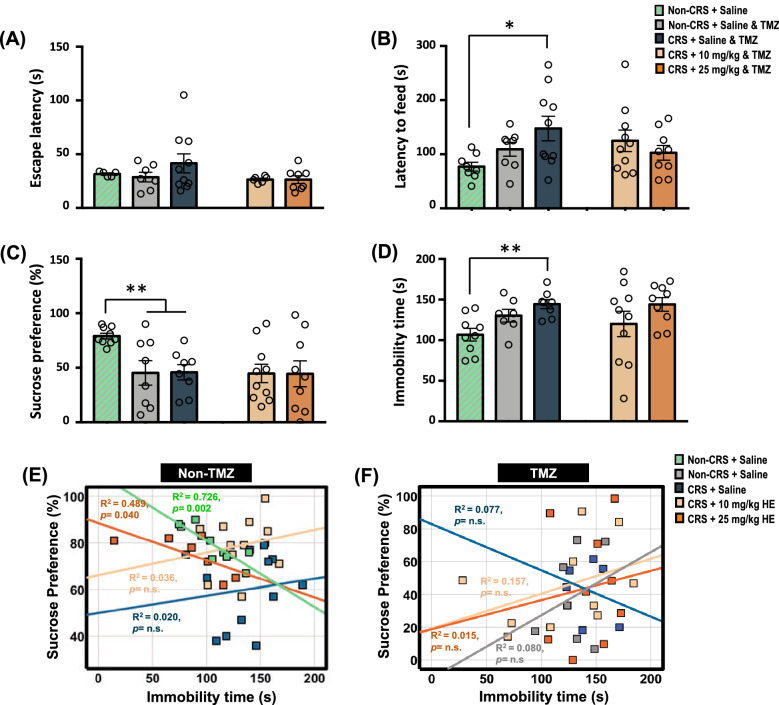
Graphical representation of the behavioural data in TMZ experimental groups. No significant differences were found in all the behavioural testing for the assessment of anxiety-like behaviour including cage emergence test (**A**) and novelty suppressed feeding test (**B**), and depressive-like behaviour including sucrose preference test (**C**) and tail suspension test (**D**). Scatter plots of the correlations between the depression-related behavioural tests in the non-TMZ (**E**) and TMZ experimental groups (**F**). Notably, in the non-TMZ experimental group, there were significant correlations between the sucrose preference and immobility time in CRS + 10 mg/kg HE, CRS + 25 mg/kg HE, and non + CRS control groups. No correlation was found between the sucrose preference and immobility time in CRS + saline group or all TMZ experimental groups. *p* ≤ 0.05 considered statistically significant

In view of the observed molecular mechanisms underlying the antidepressant-like effects of HE, we next evaluated if neurogenesis mediated the effects of HE on depression. Animals subjected to the CRS protocol were administered a neurogenesis blocker TMZ together with HE treatments before being subjected to behavioural testing. Although no significant difference was observed in cage emergence test (F_(2,19)_ = 0.929, p = n.s.) (Fig. 8A), the one-way ANOVA of the saline-treated groups revealed significant group effect in novelty suppressed feeding test (F_(2,23)_ = 4.38, p = 0.024) (Fig. 8B), sucrose preference test (F_(2,22)_ = 6.90, p = 0.005) (Fig. 8C), and tail suspension test (F_(2,21)_ = 7.41, p = 0.004) (Fig. 8D). Further analysis verified the effects of TMZ administration as TMZ-treated CRS + Saline group, which exhibited a significantly higher degree of depressive behaviours, as indicated by a longer latency to feed in novelty suppressed feeding test and longer immobility time in the tail suspension test. Our results also showed that TMZ completely abolished the anxiolytic and antidepressant-like effects of HE*.* One-way ANOVA showed that there were no statistically significant differences between all the TMZ groups as determined by the behavioural tests, including cage emergence test (F_(3, 27)_ = 1.51, *p* = n.s.), novelty suppressed feeding test (F_(3, 33)_ = 1.20, *p* = n.s.), sucrose preference test (F_(3, 31)_ = 0.004, *p* = n.s.), and tail suspension test (F_(3, 30)_ = 1.22, *p* = n.s.). These findings suggest that neurogenesis may be a plausible mechanism by which HE exerts anxiolytic and antidepressant effects. Next, we further analysed the correlation of the behavioural parameters to study the effects of neurogenesis blockade. In the non-TMZ experimental group, Pearson correlation analysis showed significant correlations between the sucrose preference percentage and immobility time in the tail suspension test in CRS + 25 mg/kg HE group (r^2^ = 0.489, *p* = 0.040) and the non-CRS control group (r^2^ = 0.726, *p* = 0.002), indicating 25 mg/kg HE potentially rescues behavioural despair due to CRS compared with normal non-CRS control animals (Fig. 8E; Table 2). No correlation was found between the sucrose preference percentage and immobility time in CRS + 10 mg/kg HE group (r^2^ = 0.036, *p* = n.s.) and CRS + saline group (r^2^ = 0.020, *p* = n.s.), suggesting a higher dose of HE is required to rescue behavioural despair induced by CRS. As expected, no significant correlation was found between the behavioural data in all TMZ experimental groups (all r^2^ < 0.157 *p* = n.s.) (Fig. 8F), indicating the blockade of neurogenesis by TMZ disrupts the anxiolytic and antidepressant-like effects of HE.

### HE reduces neuroinflammation via a neurogenesis-dependent mechanism in the animal model of depression

**Fig. 9:**
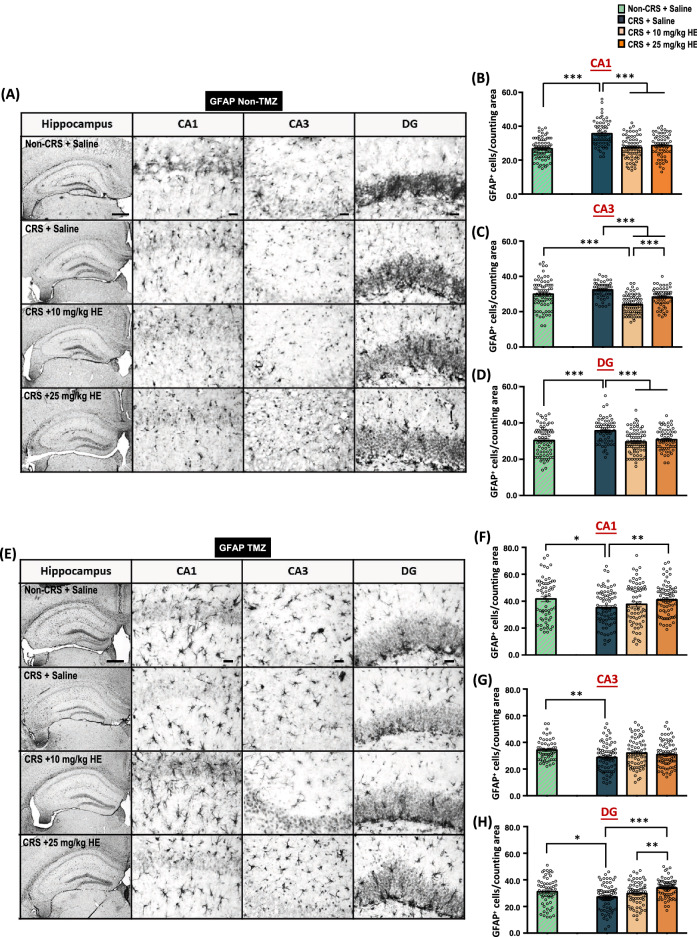
Immunohistochemical analysis of GFAP (astrocyte marker) in the hippocampus of non-CRS + saline, CRS + saline, CRS + 10 mg/kg HE, and CRS + 25 mg/kg HE group. Scale bar: 200 µm (hippocampus) and 50 µm (subregions) (**A**). The degree of astrocyte activation was examined in the CA1 (**B**), CA3 (**C**), and DG (**D**), separately. Notably, the CRS + saline group exhibited increased astrocyte activation in the CA1 and DG, which was suppressed by 10 and 25 mg/kg HE. To probe whether the observed suppression was mediated by hippocampal neurogenesis, the hippocampus of TMZ-treated animals was stained with GFAP in the same subregions. TMZ administration abolished the suppressive effects of HE (**E–H**)

Activation of astrocytes and subsequently increased levels of inflammatory cytokines are commonly observed in depressed patients and preclinical animal models of depression [58–60]. To examine whether CRS induces microgliosis or astrogliosis in CRS animals, and if so, whether HE exerts antidepressant effect via neuroinflammation-dependent pathways, brain sections were stained with glial fibrillary acidic protein (GFAP). One-way ANOVA of GFAP expression revealed significant group effects for CA1 (F_(2,191)_ = 25.746, *p* < 0.001), CA3 (F_(2,191)_ = 45.184, *p* < 0.001), and DG (F_(2,191)_ = 16.622, p < 0.001) in the dorsal hippocampus of animals in non-TMZ group. Multiple comparisons showed that GFAP expression was increased in the CA1 and DG of the CRS + saline group compared with the non-CRS control group. Both 10 mg/kg (all subregions: *p* < 0.001) and 25 mg/kg HE (all subregions: *p* < 0.001) groups exhibited reduced numbers of GFAP^+^ cells in all subregions of the dorsal hippocampus compared with the CRS + saline group. In particular, 10 mg/kg HE induced a stronger GFAP reduction in the CA3 compared with 25 mg/kg HE (Fig. 9A–D). The TMZ treatment in CRS + saline animals resulted in lower GFAP^+^ cell count compared with non-CRS control animals. Moreover, significant group effects were observed in CA1 (F_(2,216)_ = 3.54, *p* = 0.031) and DG (F_(2,211)_ = 12.46, *p* < 0.001), but not in CA3 (F_(2,216)_ = 1.97, *p* = n.s.). Multiple comparisons revealed that TMZ could effectively abolish the influence of 10 mg/kg HE*,* but not 25 mg/kg HE, on hippocampal GFAP expression, as indicated by increased GFAP^+^ cell count in the CA1 and DG of 25 mg/kg HE group (Fig. 9E–H). Taken together, these data suggest that the anti-neuroinflammatory activity of HE may be mediated by a neurogenesis mechanism.

## Discussion

*Hericium erinaceus* is a well-known medicinal mushroom that possesses potent antioxidant properties. Endogenous and exogenous antioxidants and other phytochemicals can act to maintain or re-establish redox homeostasis in response to oxidative damage [37, 61]. Our findings showed that the total polyphenol content in HE was 2.1- and 1.5-fold lower than in the aqueous extract of HE originating from Korea (4.75 mg GAE/g) and China (3.33 mg GAE/g), respectively [62]. On the other hand, the total flavonoid content in HE was 2.1-fold higher than in the HE aqueous extract (0.34 ± 0.01 mg CE/g) reported by Kim et al. (2013) [63], and 3.7-fold higher than in the HE aqueous extract grown on germinated green rice (HEGR-W) (0.197 ± 0.004 mg CE/g) reported by Lee et al. (2017) [64].

Yildiz et al. (2015) demonstrated that a high phenolic content contributes to potent antioxidant activities in numerous medicinal mushrooms including HE [65]. We found the total antioxidant capacity of HE was 1.5-fold lower than in the HE aqueous extract (11.93 mg AAE/g) reported by Charumathy et al. (2016) [66]. Nevertheless, our results also demonstrated that HE had higher DPPH free radical scavenging activity, with a 1.5-fold lower EC_50_ value compared to that in the hot water extract of HE (2.13 mg/mL) from fresh fruiting bodies [66]. Li et al. (2012) showed a water subfraction of HE methanol extract had an EC_50_ value of 4.8 mg/mL, which was 3.3-fold higher than in HE [67]. This is in agreement with HE water and ethanol extracts reported by Jiang et al. (2016), which exhibited the highest antioxidant activities among other solvent extracts, indicating the antioxidant activity was attributed to the polar ingredients [68]. Our results also demonstrated the FRAP value of HE was 2.7-fold higher than in a water subfraction from a methanol extract (19.46 ± 1.05 µmol FeSO_4_.7H_2_O equivalents/g) of fresh HE fruiting bodies reported by Li et al. (2012) [67]. The FRAP value of HE was 5.5-fold higher than in an acetone/water/acetic acid (ratio of 70:29.5:0.5) extract (9.6 ± 0.6 µmol FeSO_4_.7H_2_O equivalent/g) from HE fruiting bodies [69]. This finding was in agreement with water and hot water extracts that possessed higher antioxidant properties than acetone and ethanol extracts [70].

Our previous findings showed that HE consists of two major compounds, namely adenosine and herierin III. Adenosine was isolated as a white powder with a molecular formula of C_10_H_13_N_5_O_4_. It is an endogenous nucleoside classified under glycosylamines with an adenine attached to a ribose. Herierin III was isolated as a colourless oil with a molecular formula of C_8_H_10_O_4_. It is a heterocyclic compound classified under pyrones [37].

Herierin IV was first discovered by Qian et al. [71] from HE mycelia cultivated on solid media. The yield of herierin IV was 0.005%, which is 0.022-fold lower than the amount (0.227%) obtained from ethyl acetate extract of HE mycelia [72]. The yield was also found to be 0.25-fold lower than the amount (0.02%) from methanol extract of HE mycelia [73]. Herierin IV is a heterocyclic aromatic compound classified as a pyrone. The health promoting effects of this compound have not been previously reported [74].

In a recent study by Chiu et al. (2018), an ethanol extract of erinacine A enriched-HE mycelium was found to revert the depression-like behaviour via the activation of BDNF/TrkB/PI3K/Akt/GSK-3β pathways and inhibition of NF-κB signalling in mice challenged by repeated restraint stress [23]. In addition, our study suggests a contributory role of neurogenesis in the amelioration of depressive-like behaviours using an established TMZ-treatment protocol. Hippocampal neurogenesis was pharmacologically blocked with TMZ. Furthermore, HE used in this study is a trademark registered with the Intellectual Property Corporation of Malaysia (No. 2018001586) and is classified as a dietary supplement for humans. The antidepressant effects have been attributed to the major compounds found in HE, namely adenosine, herierin III, and herierin IV.

In the current study, we used the network pharmacology approach to predict the potential genes and pathways by which HE mediates its effects. We performed GO enrichment and KEGG pathway analyses, which showed that activity of HE compounds were associated with various neurochemical processes that are dysregulated in depression, including MAPK, IL-17, TNF and neurotrophin signalling. This observation may have profound implications. MAPK is consistently reported to be an essential molecule in the pathophysiology of depression. Interestingly, Duric et al. [75] demonstrated that MAPK cascade inhibition by upregulating MAPK phosphatase (MKP-1) expression was sufficient to induce depressive-like behaviours in rodents. This finding was further confirmed in post-mortem samples, where augmented MKP-1 expression was detected in the hippocampus of a depressed subject [75]. On the other hand, elevated peripheral IL and TNF levels were reported in patients with depression [76]. Accumulating data suggests that specific classes of antidepressants indeed normalize dysregulated pro-inflammatory cytokines in depression [77], possibly validating the inflammatory hypothesis of depression, which could be targeted in the development of antidepressants. Neurotrophic factors such as BDNF and NGF have also attracted attention in terms of their potential as antidepressants [53, 78]. To further illustrate the complexity of the molecular pathology of depression, MAPK signalling also serves as an intermediate component downstream of the BDNF-TrkB cascade. These findings, together with the results from GO and KEGG pathway enrichment analyses, show that the aforementioned pathways are tightly linked with the pathological mechanism of depression.

Common symptoms of clinical depressive disorders include anxiety, low mood, decreased appetite, lack of motivation, anhedonia, tiredness, and hopelessness [2, 3]. The CRS animal model of depression generated robust depressive-like behavioural phenotypes. After 2 weeks of 6 h consecutive restraint stress, animals with CRS exhibited early latency to escape from the aversive cage and higher latency to feed in the brightly lit open field compared with non-CRS control animals, indicating increased levels of anxiety. Furthermore, the CRS + saline group showed significantly reduced sucrose intake and increased immobility time in the tail suspension test compared with the non-CRS control group, suggesting anhedonia and behavioural despair induced by CRS. The anxiety and depression-related behavioural results showed marked differences between the behavioural phenotypes of the non-CRS control and CRS + saline groups, validating the CRS-induced animal model of depression.

The results revealed that CRS animals receiving the 25 mg/kg HE treatment had longer latency of escape from the aversive cage compared with CRS + saline animals, suggesting 25 mg/kg HE has potential anxiolytic effects. In the novelty suppressed feeding test, CRS groups receiving 10 or 25 mg/kg HE treatments had shorter latency to feed compared with the CRS + saline group, suggesting both HE treatment dosages improved anxiety and motivation for food intake. Additionally, significantly higher sucrose consumption was demonstrated in both HE treatment groups compared with the CRS + saline group, indicating HE has anti-anhedonic effects. The CRS animals receiving 25 mg/kg HE exhibited a lower immobility time in the tail suspension test, indicating 25 mg/kg HE could rescue behavioural despair in the CRS animals. Overall, the behavioural findings demonstrated that HE can ameliorate depressive-like behaviours in the CRS animal model of depression.

Previous findings showed that depression was associated with neuronal atrophy and loss of neurones, especially in the hippocampus [79]. In this study, we focused on the neurogenesis-related changes in the dorsal hippocampus involved in the antidepressant-like effects of HE. Gene assay results revealed that *Bdnf* transcript level in the dorsal hippocampus was significantly lower in the CRS + saline group compared with the non-CRS control group, which is consistent with previous reports that demonstrated an association between decreased BDNF expression and depressive-like symptoms [80, 81]. The hypothesis that BDNF replenishment mediates antidepressant effects supports the role of BDNF in depressive disorders, as BDNF was found to possibly play a role in the therapeutic mechanism of antidepressants [80, 82]. We found both the CRS + 10 mg/kg HE and CRS + 25 mg/kg HE treatment groups exhibited significantly increased neuroplasticity- and neurogenesis-related gene expressions, including *Bdnf, Syp, Nestin,* and *Psd-95*. Interestingly, gene expressions of *TrkB* and *Dcx* were significantly elevated in the 10 mg/kg HE group, but not in 25 mg/kg HE group. Synaptophysin is a synaptic marker of neuroendocrine cells that participates in synaptic transmission, whereas nestin and doublecortin are common markers for neurogenesis and are generally expressed in developing neuronal progenitor cells during adult neurogenesis [83, 84]. In our study, HE restored *BDNF* expression and upregulated *TrkB, Syp, Nestin,* and *Dcx,* suggesting potential antidepressant-like effects in CRS animals through the enhancement of BDNF/TrkB signalling, eventually leading to neurogenesis.

To support the findings of the gene expression study, we performed Western blot analyses to examine expression levels of neuroplasticity-related proteins. The CRS + saline group showed remarkably lower TrkB, pTrkB, pCREB, and pERK1/2 protein expressions compared with the non-CRS control group. Decreased pCREB and pERK2 expression levels have been previously reported in the hippocampus and prefrontal cortex of stressed animals [85, 86]. There were no significant differences in the expression levels of pro-BDNF, CREB, and ERK1/2 proteins between CRS + saline and non-CRS control groups, suggesting the expression levels of these proteins are not affected by CRS. However, *Bdnf* gene expression level was decreased in CRS animals. This may be attributed to the timeline of the animal sacrifice, in which transcribed mRNA had not been translated into proteins. Indeed, expression levels of BDNF, TrkB, and CREB proteins were reported to be decreased in post-mortem brains of suicide victims [87]. Additionally, lower plasma BDNF level was found to be associated with suicidal behaviour in major depressive disorder [88].

Our results showed that HE had robust antidepressant effects by reversing the reduced protein expression levels of mature BDNF and pCREB at 25 mg/kg HE, and rescued pTrkB at both 10 and 25 mg/kg HE. Although mature BDNF protein expression was not remarkably increased in the CRS + 10 mg/kg HE group compared to the CRS + saline group, *Bdnf* mRNA level was significantly increased in both 10 and 25 mg/kg HE treatment groups. This data suggests that 10 mg/kg HE might require a longer period to increase the BDNF protein level in the dorsal hippocampus of CRS animals compared with 25 mg/kg HE. A previous study demonstrated that 200 mg/kg HE ethanol extract increased both BDNF and TrkB expression levels in mice after 4 weeks of treatment [23]. An increased hippocampal BDNF level is often reported in animals receiving antidepressants [89]*.* Furthermore, BDNF expression was also found to be elevated in the DG in the hippocampus at post-mortem in subjects on antidepressant treatments [87]*.* We found no changes in the protein expression level of pro-BDNF in the dorsal hippocampus of CRS animals treated with HE, which is in contrast to a previous clinical study that found 8 weeks of HE treatment significantly increased circulating pro-BDNF levels without significantly changing circulating BDNF levels [25]. This discrepancy could be due to the different treatment lengths or the different tissues collected for analysis. Based on the hypothesis that HE can modulate the protein expression levels of pro-BDNF and mature BDNF in different tissues, consideration should be given to the brain region or treatment length in future studies, as these proteins are well known to be associated with antidepressant-like effects. Additionally, many studies have identified the bioactive compounds of HE that stimulate NGF expression [26, 29–35], however, the bioactive compounds that contribute to increased BDNF expression have yet to be identified.

Interestingly, TrkB, CREB, and pERK protein expressions were elevated in CRS animals receiving 10 mg/kg HE, but not with 25 mg/kg HE. Similarly, previous studies demonstrated that antidepressants could restore the decreased expressions of pCREB and pERK2 in stressed animals, but not the expression of CREB and ERK [85, 86]. Therefore, it appears that the antidepressant effects of HE are specifically induced through the activation of TrkB, ERK, and CREB to elevate protein levels of pTrkB, pERK, and pCREB. These results suggest that the antidepressant-like effects of HE could be possibly mediated through a BDNF/TrkB/CREB signalling pathway, leading to hippocampal neurogenesis. Furthermore, our findings showed that HE stimulated the synthesis of BDNF. Overexpressed BDNF activates TrkB through phosphorylation and triggers the downstream MEK-ERK signalling cascade. This cascade leads to the phosphorylation of CREB, which is a major transcription factor regulating the expression of neuroplasticity-related genes, eventually promoting neurogenesis and neurite outgrowth [53]. Increased activities in the CREB cascade in the hippocampus have been shown to increase neurogenesis of dentate granule cell progenitors and dendritic length and branching, leading to an antidepressant-like response [90, 91]. Morphological analysis of neurogenesis marker BrdU showed increased newborn cell proliferation in the 10 mg/kg HE group, confirming its neurogenic effects. Indeed, these results are in line with previous reports that demonstrated antidepressants could selectively enhance cell proliferation in the DG [92]. However, we found decreased numbers of cells stained with both NeuN and BrdU in CRS animals receiving 25 mg/kg HE. This may imply that a chronic high dose of 25 mg/kg HE may delay or reduce cells differentiation into mature neurones, and that the antidepressant effects observed at 10 mg/kg HE were attributed to neurogenesis-independent mechanisms. The unaffected BrdU^+^/NeuN^+^ cell count in the 10 mg/kg HE group suggests the involvement of other cell types mediating the therapeutic effects of HE, such as neural stem cells or glial cells. Further histological investigations on the involvement of neural stem cells and immature neurones are highly warranted to examine the role of early phase neurogenesis in the antidepressant effects of HE.

The results of the gene and protein expression study and immunofluorescence staining suggest that the mechanism of antidepressant-like effects of HE may be mediated through a neurogenesis-dependent pathway. To determine whether the underlying mechanism is neurogenesis-dependent, we used a neurogenesis blocker TMZ to block hippocampal neurogenesis. This approach is commonly used to evaluate if behavioural responses are mediated through neurogenesis-dependent mechanisms [93–96]. Our results found no significant differences in all the behavioural tests among all TMZ-treated groups, implying the administration of the neurogenesis blocker completely abolished the antidepressant-like effects of HE. This result supports our hypothesis that the antidepressant-like effects of HE are mediated through a neurogenesis-dependent mechanism, which is in line with the previous finding of enhanced hippocampal neurogenesis after 4 weeks of 60 mg/kg HE [22]. Additionally, several bioactive compounds isolated from HE*,* such as hericenones [30], erinacines [97], ergosterol peroxide, cerevisterol, and 3β,5α,9α-trihydroxy-ergosta-7,22-dien-6-one39 [35], have been found to induce NGF-activity and enhance neuritogenesis, both of which were reported to be involved in alleviating depressive-like symptoms [98–103]. To further investigate the effects of HE, we conducted a correlation study of the depression-related behavioural tests. We found a significant positive correlation between the sucrose preference and immobility time in both CRS + 10 mg/kg HE and CRS + 25 mg/kg HE groups and the non-CRS control group, but not in the CRS + saline group, suggesting HE rescues the behavioural despair in CRS animals. In contrast, no positive correlation was found for all TMZ groups, implying that neurogenesis blocker TMZ abolished the antidepressant effects of HE*.* We further examined the effects on neuroinflammation by analysing GFAP expression. We previously proposed that the anti-inflammatory activity of HE was part of multifactorial mechanisms underlying the therapeutic effects in depression [28]. The anti-inflammatory effects of HE have been widely reported, including reducing pro-inflammatory cytokines, increasing anti-inflammatory cytokines, and mitigating oxidative stress [20, 23]. Zhang et al. [58] used a transgenic animal model that is prone to astrocyte activation to show hyperactivate astrocytes contributed to the secretion of pro-inflammatory factors such as interleukin-6 and tumour necrosis factor α (TNF-α), which were associated with depressive behaviour. In our study, we found that hippocampal GFAP^+^ astrocytes were reduced in CRS animals treated with 10 and 25 mg/kg HE. This result is in line with the previous finding that amycenone, a bioactive compound isolated from HE, attenuated TNF-α in an LPS-induced inflammation model of depression [21], suggesting that HE can alleviate neuroinflammation by suppressing astrocyte activation. Moreover, TMZ administration completely abolished the suppressive effects of HE, further strengthening the evidence for the neurogenesis-dependent therapeutic effects of HE.

In conclusion, our study showed that 4 weeks of intraperitoneal administration of HE alleviated depressive-like behaviours in a CRS model of depression. Taken together, the present findings showed the HE treatment induced antidepressant and anti-inflammatory effects via a neurogenesis-dependent mechanism. We found that HE rescued behavioural despair possibly through a BDNF-TrkB-CREB signalling mechanism, leading to increased hippocampal neurogenesis and decreased neuroinflammation that contribute to the antidepressant effects.

## Supplementary Information

**Additional file 1: Fig. S1.** NMR data assignments. Schematic diagram of the chemical structure of herierin IV (A). Steps involved in the isolation of HE compounds (B). **Fig. S2**. Visualization of MAPK (A) and IL-17 (B) signalling pathways. **Fig. S3**. Visualization of TNF (A) and neurotrophin (B) signalling pathways.

**Additional file 2: Table S1.** ^1^H and ^13^C NMR (600 MHz) spectroscopic data for herierin IV (**3**) in MeOH-_d4_.

## Data Availability

The datasets from and/or analysed during the current study are available from the corresponding author on reasonable request.

## Abbreviations

AKT: Protein kinase B
BDNF: Brain-derived neurotrophic factor
BrdU: Bromodeoxyuridine
CA: Cornu Ammonis
CREB: CAMP response element-binding protein
CRS: Chronic restraint stress
DCX: Doublecortin
DG: Dentate gyrus
DPPH: 2,2-Diphenyl-1-picrylhydrazyl
ERK: Extracellular signal-regulated kinase
FRAP: Ferric reducing antioxidant power
GAPDH: Glyceraldehyde 3-phosphate dehydrogenase
GFAP: Glial fibrillary acidic protein
HE: *Hericium erinaceus*
HPLC: High-performance liquid chromatography
Nes: Nestin
NeuN: Neuronal nuclei
NGF: Nerve growth factor
PSD-95: Postsynaptic density protein 95
SYN: Synapsin
TMZ: Temozolomide
TNF-α: Tumour necrosis factor α
TrkB: Tropomyosin receptor kinase B
