# Supplementary figures and images for: Neurogenesis-dependent antidepressant-like activity of Hericium erinaceus in an animal model of depression
Source: Chin Med. 2021 Dec 7;16:132. doi: 10.1186/s13020-021-00546-8 (PMC8650354; doi:10.1186/s13020-021-00546-8)

**Figure S1.**

**(A)**

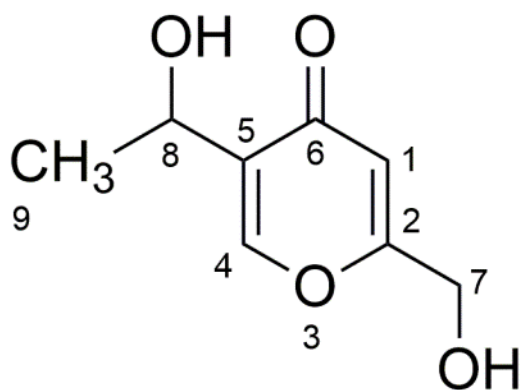

**(B)**

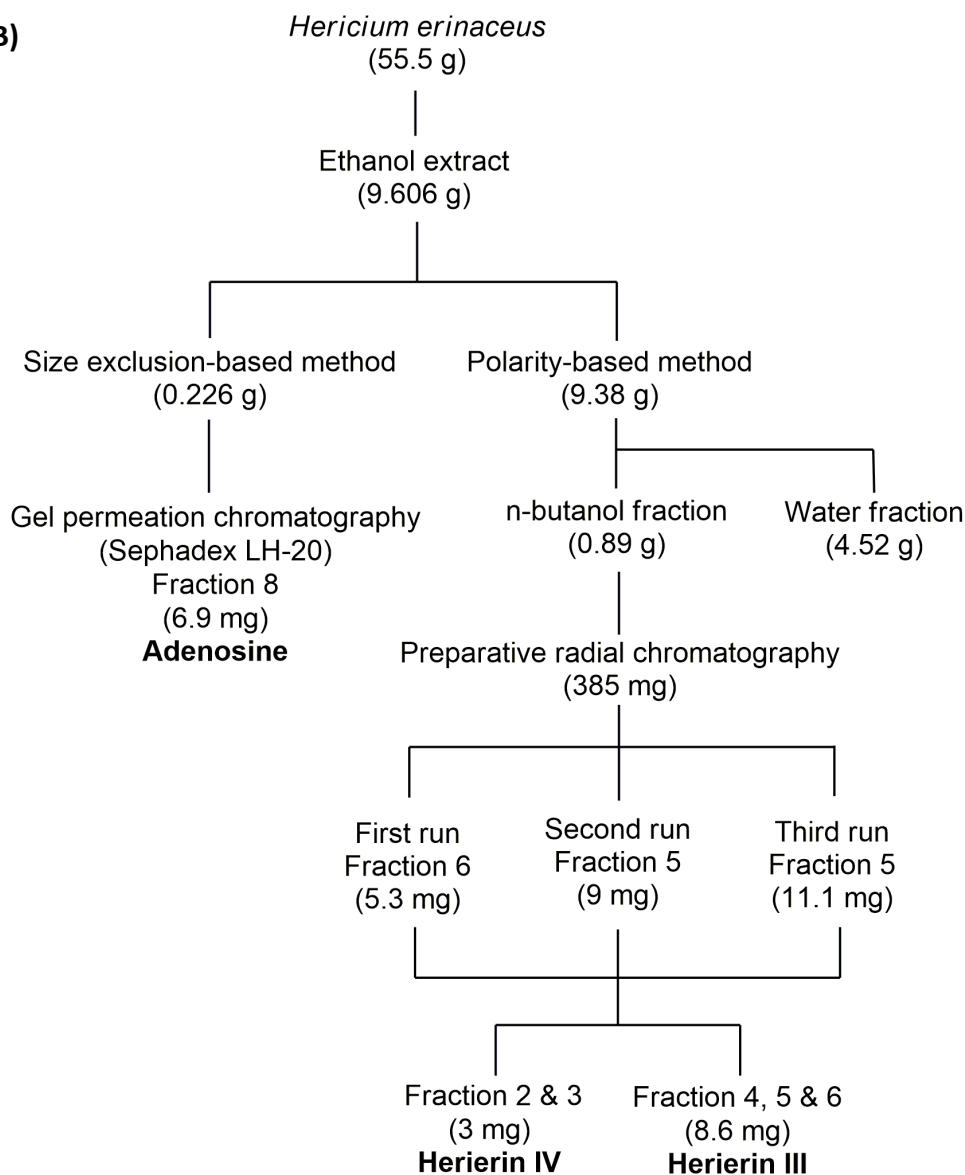

**(A)**

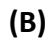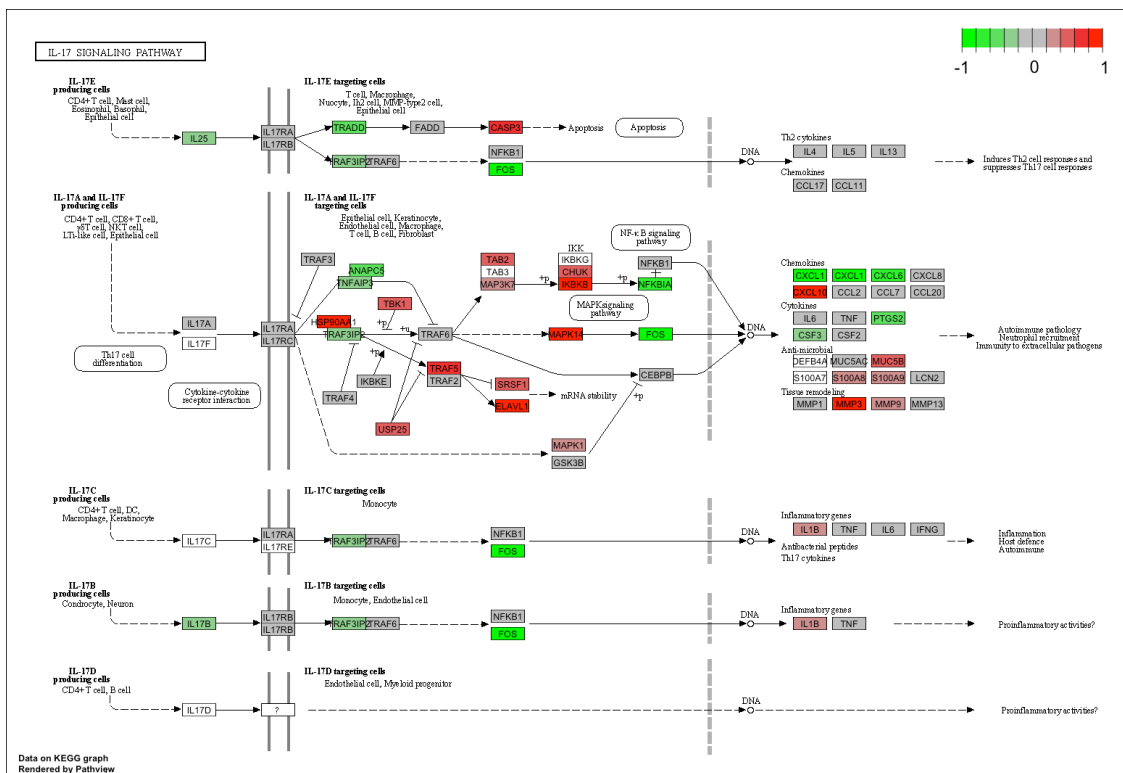

**Figure S3.**

**(A)**

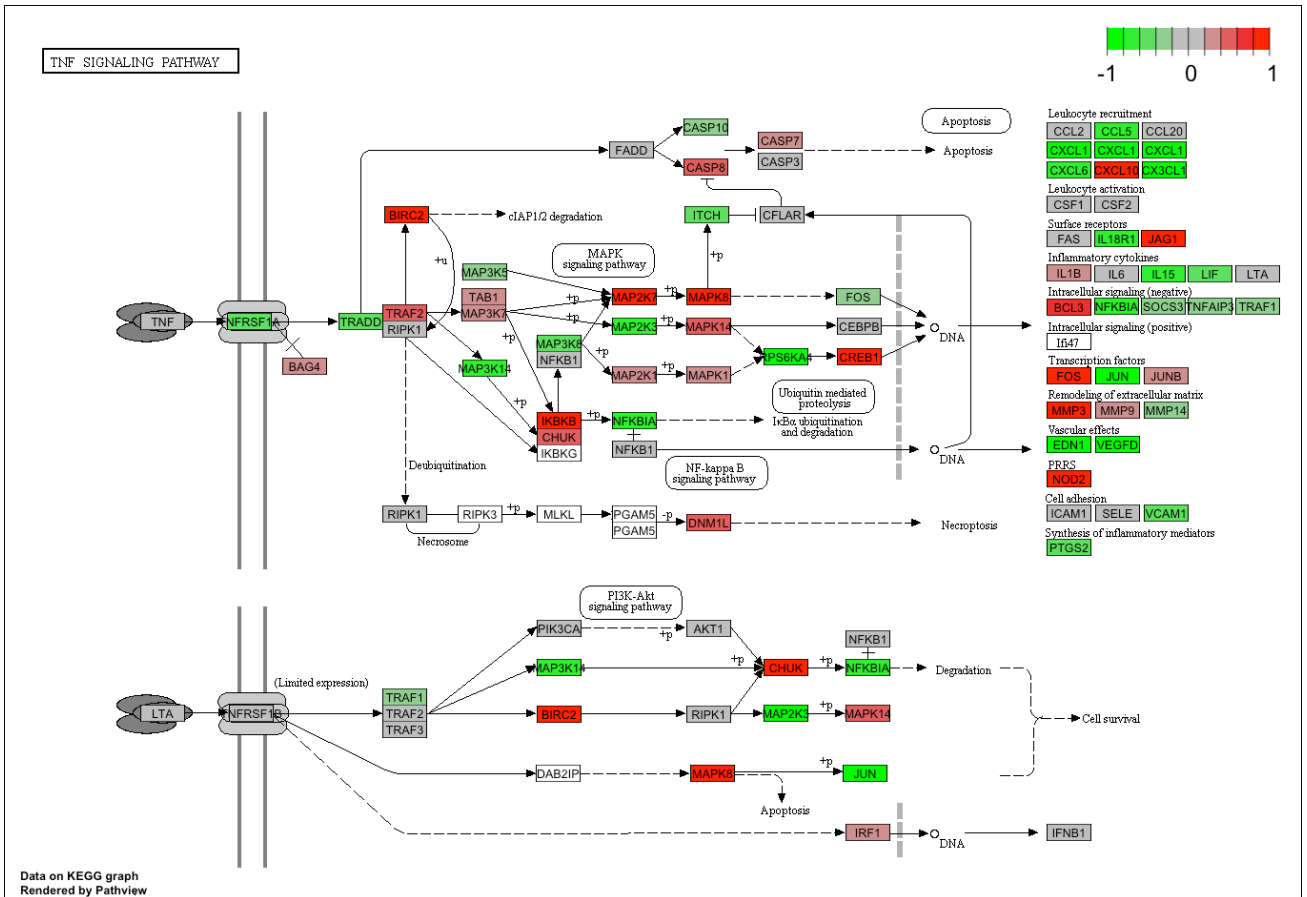

**(B)**

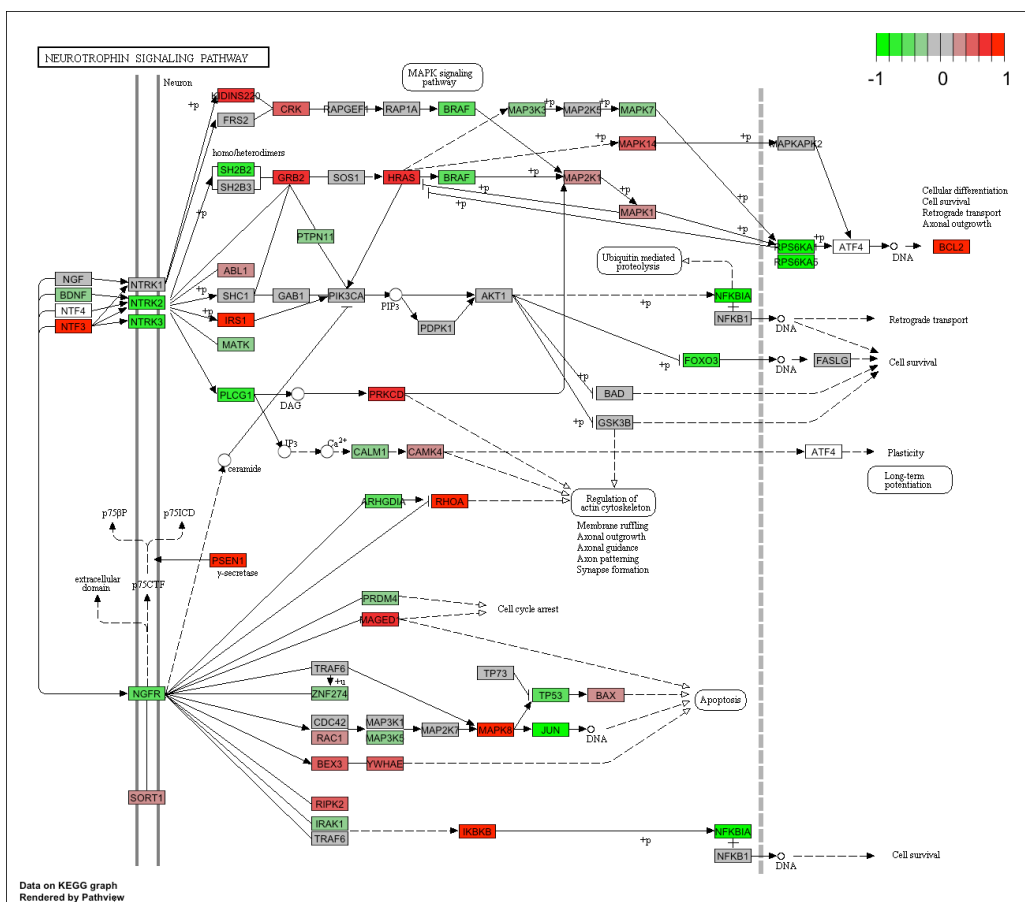

Supplement: Supplementary file 1 — Additional file 1: Fig. S1. NMR data assignments. Schematic diagram of the chemical structure of herierin IV (A). Steps involved in the isolation of HE compounds (B). Fig. S2. Visualization of MAPK (A) and IL-17 (B) signalling pathways. Fig. S3. Visualization of TNF (A) and neurotrophin (B) signalling pathways. [file 13020_2021_546_MOESM1_ESM.pdf]
