# Supplementary material for: Neurogenesis-dependent antidepressant-like activity of Hericium erinaceus in an animal model of depression
Source: Chin Med. 2021 Dec 7;16:132. doi: 10.1186/s13020-021-00546-8 (PMC8650354; doi:10.1186/s13020-021-00546-8)
Supplement: Supplementary file 2 — Additional file 2: Table S1. 1H and 13C NMR (600 MHz) spectroscopic data for herierin IV (3) in MeOH-d4. [file 13020_2021_546_MOESM2_ESM.docx]

**Supplementary Table 1.** ^1^H and ^13^C NMR (600 MHz) spectroscopic data for herierin IV (**3**) in MeOH-_d4._

| **Position** | **^1^H (ppm)** | **^13^C (ppm)** |
| --- | --- | --- |
| 1 | 6.42 s | 112.5 |
| 2 |  | 171.3 |
| 4 | 8.07 s | 171.7 |
| 5 |  | 134 |
| 6 |  | 180.8 |
| 7 | 4.64 s | 61 |
| 8 | 4.43 s | 63.6 |
| 9 | 1.38 d | 23 |
